# Editorial: 90th anniversary of the Sherrington and Adrian Nobel Prize: brain rhythms and oscillations in neural activity modulation, brain function and dysfunction

**DOI:** 10.3389/fncel.2023.1332956

**Published:** 2023-11-22

**Authors:** George C. McConnell, L. Stan Leung

**Affiliations:** ^1^Department of Biomedical Engineering, Stevens Institute of Technology, Hoboken, NJ, United States; ^2^Department of Physiology and Pharmacology, University of Western Ontario, London, ON, Canada

**Keywords:** oscillatory activity, brain rhythms, hippocampus, spectral analysis, prefrontal cortex, local field potentials (LFPs), neural dynamics, neural recording and stimulation

In 1932, Charles Scott Sherrington and Edgar Douglas Adrian received the Nobel Prize Award in Physiology or Medicine for their groundbreaking work on the function of neurons. A thorough physiological study of spinal reflex and its modulation revealed integrative properties including the concept of synapse with a synaptic delay (Sherrington, 1906). Adrian's (1942) pioneering studies used then-state-of-the-art amplification to record action potentials from single sensory fiber and electrical oscillations in the olfactory bulb.

We, George McConnell and L. Stan Leung, had the privilege to edit this Research Topic to “highlight cutting-edge research progressing our understanding of how brain rhythms and neural oscillations modulate neural response dynamics and relation of oscillations to brain function and dysfunction.” Eight articles were published in this Research Topic.

Field potentials have been instrumental in advancing the study of the dynamics and behavioral correlates of oscillations. In their article “*Theoretical considerations and supporting evidence for the primary role of source geometry on field potential amplitude and spatial extent*,” Herreras et al. reviewed the generation of field potentials in the brain focusing on mesoscopic current sources, which represent coherent synaptic activity activated in a population of neurons by a given anatomical pathway. The mesoscopic sources are revealed by the application of independent component analysis on high-density recordings in the brain. The geometry or three-dimensional distribution of a mesoscopic source determines the spatial extent and drop-off of the field potentials generated in different dimensions. Some interesting examples are a high-amplitude theta rhythm in the hilus of the dentate gyrus where there are no current sources, a theta field recorded in the lateral habenula that could be attributed to the hippocampus, and delta waves from a large cortical source module spreading to subcortical areas.

A theme common to several studies in this Research Topic was using spectral analysis to identify neural activity patterns of local field potentials (LFPs) in the hippocampus. In their article “*Visual cortical LFP in relation to the hippocampal theta rhythm in track running rats*,” Kennedy et al. revisited the topic of generation of LFPs in the primary visual cortex (VC) during running, using multichannel recordings in the VC and hippocampus of rats. Power spectral density analysis revealed that LFP power in the VC was similar to that in the hippocampus but with a lower magnitude. The current source density triggered to the maximal theta peak did not reveal clear sources and sinks in the VC. The power and frequency of theta harmonics increased with running velocity, with a similar change in the VC and hippocampus. Bicoherence analysis indicated that phase coupling between theta and theta harmonics was high in both the hippocampus and VC. However, gamma was not significantly coupled to theta in the VC of rats. The authors concluded that theta oscillations in the VC are likely due to volume conduction from the hippocampus. In their article “*Linking temporal coordination of hippocampal activity to memory function*,” Etter et al. reviewed hippocampal oscillations in learning and memory with a focus on the relative contributions of LFPs and single-neuron activity. Examples of loss of rhythmicity and gain of rhythmicity in the context of memory performance are discussed, including oscillations induced by neural stimulation therapies for Alzheimer's disease. In their article “*Development of network oscillations through adolescence in male and female rats*,” Sibilska et al. expanded our knowledge of sex differences in the maturation of oscillations in the prefrontal cortex (PFC) and hippocampus. Their study of the adolescent period in healthy rats paralleled the vulnerable age of schizophrenia prodrome in humans. In their article “*Locus coeruleus noradrenergic neurons phase-lock to PFC and hippocampal infra-slow rhythms that synchronize to behavioral events*,” Xiang et al. investigated locus coeruleus (LC) neuronal synchrony to infra-slow rhythms in the PFC and hippocampus in awake and behaving rats. The finding of phase-modulated gamma amplitude by infra-slow oscillations suggests a potential mechanism by which noradrenaline released by LC neurons could facilitate behavioral adaptation.

The dopaminergic system of the midbrain was the focus of Oberto et al. in their article “*Rhythmic oscillations in the midbrain dopaminergic nuclei in mice*,” which characterized oscillations and behavioral correlates in the ventral tegmental area (VTA) and substantia nigra pars compacta (SNc). The authors proposed future work investigating the coherence of the VTA and SNc with the striatum, PFC, and hippocampus to further investigate dopaminergic coordination of “goal-directed decision making, learning, and motor control.”

Faber et al., in their article titled “*Frequency-coded patterns of sympathetic vasomotor activity are differentially evoked by the paraventricular nucleus of the hypothalamus in the Goldblatt hypertension model*,” investigated the role of the paraventricular nucleus of the hypothalamus (PVN) in the spectral features of the renal and splanchnic sympathetic nerve activity in a rat model of neurogenic hypertension. The study highlights the utility of spectral analysis of neural recordings from the autonomic nervous system for disease models.

Agadagba et al. wrote a review article titled “*Advances in transcorneal electrical stimulation: From the eye to the brain*.” Transcorneal electrical stimulation (TES) is a non-invasive neuromodulatory technique that can lead to beneficial effects by stimulation of the retina and through the optic nerve to the brain. TES was shown to delay photoreceptor and retinal ganglion cell loss in animal models of retinitis pigmentosa (RP) and to preserve retinal function in clinical RP. Imaging in the human brain showed that the effects of TES on glucose metabolism extended beyond vision-related brain areas, and included the PFC and parahippocampal gyrus. In animals, TES can modify low-frequency brain oscillations and change functional connectivity between the primary visual cortex and PFC, with effects lasting for minutes or days after stimulation. Behavioral alteration and molecular expression studies in rodents suggest that TES can affect hippocampal and amygdala networks as well. The authors discussed the future direction for the use of TES as a non-invasive therapeutic tool for the treatment of diseases, including depression, and for improving visual attention.

Taken together, these studies updated new developments in our understanding of how neural oscillations may contribute to neural communication in both healthy and disease states ranging from hypertension and schizophrenia to neurodegenerative diseases such as Parkinson's disease and Alzheimer's disease.

## Author contributions

GM: Writing original draft, Writing review & editing. LL: Writing original draft.

## References

[B1] Adrian E. D. (1942). Olfactory reactions in the brain of the hedgehog. J. Physiol. 100, 459–473. 16991539 10.1113/jphysiol.1942.sp003955 PMC1393326

[B2] Sherrington C. S. (1906). The Integrative Action of the Nervous System. New Haven, CT: Yale University Press.

